# A multicenter prospective phase II study of postoperative hypofractionated stereotactic body radiotherapy (SBRT) in the treatment of early-stage oropharyngeal and oral cavity cancers with high risk margins: the STEREO POSTOP GORTEC 2017-03 trial

**DOI:** 10.1186/s12885-020-07231-3

**Published:** 2020-08-05

**Authors:** Julian Biau, Emilie Thivat, Corinne Millardet, Nicolas Saroul, Nathalie Pham-Dang, Ioana Molnar, Bruno Pereira, Xavier Durando, Jean Bourhis, Michel Lapeyre

**Affiliations:** 1 Department of Radiotherapy, Jean Perrin Centre, 58 rue Montalembert, BP 5026, 63011, Cedex 1 Clermont Ferrand, France; 2 INSERM U1240 IMoST, Université Clermont Auvergne, Clermont-Ferrand, France; 3 UMR 501, Centre d’Investigation Clinique, F-63001 Clermont-Ferrand, France; 4 Department of clinical research, Délégation Recherche Clinique et Innovation, Centre Jean Perrin, Clermont-Ferrand, France; 5 Medical physics department, Centre Jean Perrin, Clermont-Ferrand, France; 6 Department of Otorhinolaryngology-Head and Neck Surgery, University Hospital Center Gabriel Montpied, Clermont-Ferrand, France; 7 Department of Maxillofacial Surgery, University Hospital Center Estaing, Clermont-Ferrand, France; 8 Biostatistics Department, Délégation à la Recherche Clinique et à l’Innovation, Clermont-Ferrand University Hospital, Clermont-Ferrand, France; 9 Oncology department, Centre Jean Perrin, Clermont-Ferrand, France; 10 Department of Radiation Oncology, Centre Hospitalier Universitaire Vaudois, Lausanne, Switzerland

**Keywords:** Oropharyngeal and Oral cavity cancers, Hypofractionated stereotactic body radiotherapy (SBRT), Postoperative radiotherapy

## Abstract

**Background:**

Primary surgery is usually the mainstay treatment in early-stage oropharyngeal and oral cavity cancer. Typically, neck surgery is performed. Negative tumor margins are recommended (> 5 mm). If feasible, re-resection of any positive margin is preferred. Otherwise, postoperative radiotherapy is required. Adjuvant postoperative radiotherapy can be limited to the primary site for patients with pT1-T2 tumors and negative neck exploration. Currently, both fractionated external beam radiotherapy and brachytherapy can have a role in the postoperative management of early-stage oropharyngeal and oral cavity cancer with high risk margins. Another possible alternative could be postoperative stereotactic body radiotherapy (SBRT). The aim of this study is to evaluate postoperative SBRT in the treatment of early-stage oropharyngeal and oral cavity cancer with high risk margins.

**Methods:**

The STEREO POSTOP study is a national, open-label, non-randomized phase II trial within the GORTEC network. Patients with early-stage oropharyngeal and oral cavity cancers with high risk margins indicating the need for postoperative radiation are eligible for enrollment. SBRT consists of a total dose of 36 Gy in 6 fractions over 2 weeks. The primary endpoint is severe late toxicity defined as 2-year toxicity of grade ≥ 3 according to CTCAE V4.03 classification. The secondary endpoints include acute toxicity (≤ 3 months), local and locoregional control, disease-free and overall survival, quality of life of patients, nutritional impact and predictive factors of toxicity. The experimental design chosen is a one-step Fleming plan design without interim analysis as the primary endpoint will be evaluated at a 2-year follow-up. Ninety patients will be recruited. The study was started in January 2018 with a 4-year enrollment period and an estimated completion in January 2024.

**Discussion:**

This study is the first prospective trial to evaluate head and neck cancer postoperative SBRT in the setting of early-stage oropharyngeal and oral cavity cancers with high risk margins. SBRT is an attractive option because it delivers a highly conformal dose of radiation in a limited number of fractions (like brachytherapy but with less contraindication), with steep dose gradients resulting in reduced normal tissue irradiation and with a short overall treatment time.

**Trial registration:**

Clinicaltrials.gov: NCT03401840, registered on 17-1-2018. Identifier in French National Agency for the Safety of Medicines and Health Products (ANSM): N°ID - RCB 2017-A02058–45, registered on July 2017.

Protocol version: Version 3 dated from 25th November 2019.

## Background

Early-stage oropharyngeal and oral cavity cancers are mainly squamous cell carcinomas. The main risk factors include tobacco, alcohol and human papillomavirus (HPV) infection. Their incidence is rising [1]. Multidisciplinary management is needed. Primary surgery is usually the mainstay treatment in early-stage oropharyngeal and oral cavity cancer [2]. Typically, neck surgery is performed either by neck dissection or sentinel lymph node biopsy [3–5]. Negative tumor margins are recommended (> 5 mm) [6, 7]. If feasible, re-resection of any positive margin is preferred. Otherwise, postoperative radiotherapy is required [8–11]. Currently, both fractionated intensity-modulated radiation therapy (IMRT) and brachytherapy can have a role in the postoperative management of early-stage oropharyngeal and oral cavity cancer with high risk margins.

Brachytherapy is a highly conformal radiotherapy technique which allows high-dose delivery to small volumes within a short overall treatment time [12, 13]. However, implantation is not always technically possible: for example, for tumors that are very close to (< 5 mm) or involve bone, gingiva or the retromolar trigone, as well as parapharyngeal or nasopharyngeal extension for oropharyngeal carcinomas. Furthermore, brachytherapy necessitates highly experienced teams and appropriate infrastructure. The patient has to be hospitalized and implantation is usually carried out under general anesthesia. Goineau et al. [14] published a study concerning 112 patients treated with post-operative interstitial low dose rate (LDR) Ir-192 brachytherapy for mobile squamous cell carcinoma of the tongue. Local control rates were 79% at 2 years and 76% at 5 years. Overall survival rates were 72% at 2 years and 56% at 5 years. 22% of patients presented necrosis warranting surgery. 8% of patients had chronic pain requiring narcotics. In the study by Lapeyre et al. [15], the 2-year local control rate was 81% and 5-year overall survival rate 70% for T1/T2-N0 patients. Grade 2 and grade 3 late toxicities were 21 and 10%, respectively. Strnad et al. [16] published the largest brachytherapy study worldwide, which described a clinical trial with 385 patients. Patients were treated with interstitial pulsed-dose-rate (PDR) brachytherapy. Brachytherapy was preceded by surgery in 85% of patients. 5-year local control and overall survival were 86 and 69%, respectively. Serious late side effects, such as soft tissue or bone necrosis, were observed in 10 and 5%, respectively.

Post-operative intensity-modulated radiation therapy (IMRT) is an alternative to brachytherapy, but overall treatment time is longer (6–7 weeks) [17–21]. No randomized trial has ever compared the outcomes and toxicity profiles of post-operative brachytherapy vs IMRT for early-stage oropharyngeal and oral cavity cancers. Despite the fact that most studies mix early and advanced stages, they allow estimation of toxicity profiles. Acute mucositis and the need for long-term feeding tubes have been reported in 11–36% and 5–10% cases, respectively. The risk of severe late soft tissue necrosis (grade 3–4) varied around 2–4% and osteoradionecrosis occurred in 0–5% of patients [17–21].

Another possible alternative, which we are assessing in this trial, could be postoperative hypofractionated Stereotactic Body Radiotherapy (SBRT). SBRT delivers a single dose or a few ablative doses of radiation to extracranial tumors using advanced technology in treatment planning and image guidance [22]. To date, SBRT in head and neck cancers has been mainly studied in cases of reirradiation or as a boost for non-operated patients [23–30]. SBRT is an attractive option because it delivers a highly conformal dose of radiation in a limited number of fractions (like brachytherapy but with less contraindication), with steep dose gradients resulting in reduced normal tissue irradiation and with a short overall treatment time (6 fractions over 2 weeks in this trial vs. 30–33 fractions over 6–7 weeks for fractionated external beam IMRT).

Our phase II trial aims to evaluate postoperative SBRT in the treatment of early-stage oropharyngeal and oral cavity cancer with high risk margins with the hypothesis that the safety and efficacy profiles of postoperative SBRT will be similar to other radiotherapy techniques (brachytherapy or fractionated IMRT).

## Methods/design

### Study design

#### Design

This prospective study is designed as a national, open-label, non-randomized phase II trial and aims to evaluate toxicity and efficacy of postoperative SBRT in the treatment of early-stage oropharyngeal and oral cavity cancer with high risk margins.

The experimental plan will be performed using a Fleming’s single-stage design without interim evaluation due to the time frame of the primary end point (2-year severe toxicity).

This study has been registered on Clinicaltrials.gov (NCT03401840). The study was started in January 2018 with a 4-year enrollment period and an estimated completion in January 2024.

### Coordination

The Centre Jean Perrin is the sponsor and responsive of the coordination of the trial in cooperation with GORTEC group. Trial management, datamanagement and monitoring were delegated by the sponsor to GORTEC.

### Participating institutions

The multicenter study is currently conducted at 24 sites in France, mainly of the GORTEC network. The list of study sites is available at https://clinicaltrials.gov/ct2/show/NCT03401840.

### Objectives and endpoints

#### Main objective

The primary objective is to evaluate severe late toxicity of postoperative SBRT in the treatment of early-stage oropharyngeal and oral cavity cancer with high risk margins. The primary endpoint is 2-year late severe (grade ≥ 3) toxicity, considered as related to postoperative SBRT. An evaluation of late toxicity < 2 years could lead to a risk of underestimation of this particular situation and treatment.

The safety profile will be evaluated according to NCI CTCAE criteria V4.03.

Particular attention will be paid to the assessment of the following items that will be used for monitoring toxicity related to SBRT:

- Gastrointestinal disorders: Mucositis (soft tissue necrosis), dysphagia and dry mouth (xerostomia).
- Musculoskeletal and connective tissue disorders: Osteonecrosis of jaw.
- Skin and subcutaneous tissue disorders: Telangiectasia and skin induration.

Reporting of serious adverse events and unintended effects will be carried out according to the local regulations.

#### Secondary objectives

The secondary objectives are as follows:

- To evaluate local control. 2-year local control will be evaluated. Any local recurrence (T) documented in the area of the tumor bed will be considered as an event. The diagnosis of a local recurrence requires histological documentation.
- To evaluate locoregional control. 2-year locoregional control will be evaluated. Any local (T) or lymph node (N) (positive nodes in the ipsilateral or contralateral neck) recurrence will be considered as an event (as determined by clinical examination and/or imaging assessment showing suspicion or pathological confirmation).
- To evaluate acute toxicity profiles: acute toxicity is any ≤3-month severe toxicity (grade ≥ 3) related to SBRT according to NCI CTCAE criteria V4.03 (Cf Safety profile evaluation §3.1).
- To evaluate disease-free survival (DFS): 2-year DFS rate. DFS is defined as time from randomization to the date of first occurrence of any locoregional recurrence, distant progression or death from any cause.
- To evaluate overall survival (OS): 2-year OS rate. OS is defined as time from randomization to death from any cause.
- To evaluate quality of life of patients (QoL). QoL will be evaluated by the EORTC QLQ-C30 and H&N35 questionnaires at inclusion, 1 month, 1 year and 2 years post-SBRT.
- To evaluate nutritional impact: weight assessment at inclusion, during SBRT and then 1 week, 1 month, 3 months and every 3 months until 2 years post radiotherapy; evaluation of feeding tube use. Weight loss will be evaluated according to NCI CTCAE criteria V4.03.
- To determine predictive factors of toxicity: clinical and/or dosimetric factors associated with 2-year severe toxicities.

#### Exploratory objectives

Exploratory objectives include the evaluation of the dosimetric impact of adding non-coplanar arcs to the volumetric modulated arc therapy (VMAT) technique on a Novalis-type accelerator, and studying the dose-toxicity relationship on the first 10 patients treated with this technique.

### Study population

#### Inclusion criteria

- Operated squamous cell carcinoma of the oral cavity (lips excepted) or oropharynx
- pT1 or pT2
- Indication of postoperative tumor site irradiation (confirmed by multidisciplinary tumor board) with at least one of the following criteria:
- positive R1 margin (re-resection not proposed)
- close margin < 5 mm (re-resection not proposed)
- margin estimated at risk, with uncertain pathological margin (re-resection not proposed)
- N0 after surgical treatment of the neck (neck dissection or sentinel lymph node biopsy) or pN1 without extracapsular extension (carcinological neck dissection)
- Age ≥ 18 years
- ECOG status ≤2
- Written signed informed consent before any specific procedure of the protocol
- Affiliation to a social security scheme or beneficiary of such a scheme

#### Exclusion criteria

- Other histology than squamous cell carcinoma.
- pT3 or pT4.
- pT2 > 3 cm and R1 with concurrent chemoradiotherapy decided in multidisciplinary tumor board
- Lymphovascular invasion justifying neck irradiation
- Neck irradiation decided in multidisciplinary tumor board
- Lack of at least one of the following elements:
- pre-operative medical imaging (CT scan or MRI)
- endoscopy report
- surgery report
- pathological report
- Prior radiotherapy to the head and neck area
- Distant metastasis
- Pregnant or nursing (lactating) women
- Women or men of childbearing age not taking adequate contraceptive measures
- Participation in another investigational study within 4 weeks prior to inclusion
- History of other malignancy within 5 years prior to enrollment except for basal cell carcinoma of the skin or carcinoma in situ of the cervix
- Persons deprived of their liberty, under guardianship or curatorship, or unable to follow the trial for geographic, social or psychological reasons

### Interventions – stereotactic body radiotherapy (SBRT)

#### Facility, equipment and quality assurance of radiotherapy

SBRT modality is left free to participating institutions in function of their equipment. Dedicated stereotactic linear accelerators or adapted linear accelerators are allowed. Participating institutions must comply with the Quality Assurance of Radiotherapy requirements and procedures. All centers should perform a benchmark case procedure prior to authorization. This is a two-step procedure that contains i) a delineation and ii) planning exercise according to the protocol of a patient case that will be provided. The benchmark case will be centrally reviewed by the Quality Assurance committee of the trial. Sites that do not conform to the requirements of the audit will not be allowed to participate. A Quality Assurance check will be performed retrospectively on all patients enrolled at each site. This retrospective check will be performed as soon as possible up to a maximum of 4 months after treatment to allow for major corrections if needed for future enrolled patients.

#### Dental examination

All patients receiving SBRT should have an oral and dental examination, including a clinical and radiological examination. When indicated, extraction of dental elements should be carried out. The interval between extractions and start of SBRT should be at least 10 to 14 days. Adequate dental care (including daily fluorine application if necessary) should be recommended to all patients, at least during follow-up.

#### Patient position and data acquisition

All patients will be irradiated in a supine position. Immobilization devices such as stereotactic customized masks will be used to secure the accuracy and reproducibility of patients’ positioning during SBRT. For all patients, Planning Computed Tomography (Planning-CT), using a set of slices extending from the level of the base of skull to the lower border of the clavicle, will be required. Slice thicknesses of 1–1.25 mm will be used. To enhance vascular and soft tissue contrast and to facilitate delineation of both target volumes and organs at risk (OARs), the use of intravenous contrast enhancement is mandatory (except in case of a contra-indication).

#### Volume definition

### Delineation of the primary tumor clinical target volume (CTV)

Before starting the delineation, it is necessary to analyze the preoperative data: the diagram of the initial tumor, pre-operative medical imaging (CT scan +/− MRI +/− TEP) and endoscopy, and the surgery and pathological reports. The second step consists of revising the patients’ clinical evaluation because modifications can appear between surgery and planning-CT. A matching with the preoperative imaging can be used to help the delineation of the CTV. The CTV corresponds to the initial tumor bed including the positive or close margins with a margin from 5 to 10 mm according to the anatomical barriers and the spread zones. In case of flap reconstruction, CTV will also include the junction of the normal tissue/flap + 5 mm proximity flap.

### Determination of the planning target volume (PTV)

A set-up margin will be implemented around each CTV to take into account patient set-up uncertainties. This margin will have to be selected by each participating center depending on their equipment, irradiation techniques and experiences. Typically, for patients immobilized with a stereotactic device, a 2-mm margin appears adequate.

### Delineation of organs et risk (OAR) and planning organ at risk volume (PRV)

The delineation includes different OAR according to Brouwer et al. [31]: the spinal cord, the spinal canal, the brainstem, the parotid glands, the mandible, the lips, the pharyngeal constrictor muscle, the submandibular glands, the carotid arteries, the cochlea, the submandibular glands, the buccal mucosae and the supraglottic larynx. Additional normal structures or ‘avoidance structures’ may be delineated as an aid to the optimization process in particular to avoid hot spots outside of the PTV. This will be left to the discretion of the treating physicians and their medical physicists. A set-up margin will be added to the spinal cord, the brainstem, the cochlea and the carotid arteries to take into account patient set-up uncertainties. This margin will have to be selected by each participating center depending on their equipment, irradiation techniques and experience. Typically, for patients immobilized with a stereotactic device, a 2-mm margin appears adequate.

#### Dose prescription, specification and reporting in the PTV and overall treatment time

Prescription isodose lines are chosen to at least encompass ≥95% of the PTV, with no more than 20% of any PTV receiving doses > 110% of the prescribed dose, no more than 2% of any PTV receiving < 93% of the prescribed dose, and no more than 5% of any normal tissue receiving doses in excess of 107% of the primary PTV dose. The dose of 36 Gy in 6 fractions over 11–13 days seems to be the most appropriate schedule for this specific post-operative situation in terms of benefit/risk ratio. To determine biologically effective doses (BED), we used the following formula to take into account tumoral doubling time (Tp), overall treatment time and the cellular repopulation coefficient (Tk) [32, 33]:

$$ \mathrm{BED}= nd\left(1+\frac{d}{\alpha /\beta}\right)-\frac{Ln2\left(T-{T}_k\right)}{\alpha {T}_p} $$

With this model, we obtained a BED_10_ of 64.2 Gy for the tumor (equivalent to a BED_10_ of 60 Gy in 30 fractions); a BED_10_ of 54.4Gy for early effects (equivalent to a BED_10_ of 74 Gy in 37 fractions); and a BED_3_ of 108 Gy for late effects (equivalent to a BED_3_ of 66 Gy in 33 fractions).

Patients will preferentially be treated with the first fraction given on a Monday. The 6 fractions will be delivered in 11–13 days, 3 fractions per week. A minimum of 36 h between fractions is required.

Dose-volume constraints will be used for both dose specification and dose reporting in PTV and PRV/OAR.

#### Treatment planning

For linear accelerators, field arrangements are left to the discretion of the medical physicists to produce an optimal dose distribution matching the dose-volume constraints for PTV, PRV and OAR. Non-coplanar field arrangements are allowed, but beam directions through the eyes are not allowed unless completely unavoidable. All field entrance and exits should be within the planning CT range in order to avoid any inadequate dose calculations.

#### Time interval between surgery and SBRT

Time interval between surgery and SBRT: within 6 weeks, maximum 7 weeks.

#### Treatment verification and accuracy

Online review of the optimal patient repositioning system will systematically be performed before each fraction according to each centre’s policy and equipment (CBCT – KV/KV – Exactrac, etc.). Any necessary offset correction will be applied.

#### Treatment interruptions / modifications

No modifications (major deviations) will be permitted with regard to the target volume selection and delineation, the radiation dose prescriptions and the overall treatment time. Local investigators will carefully follow their patients during treatment and take all adequate measures to avoid any interruption and/or modification of the total dose. It is, however, the responsibility of the local investigator to interrupt the treatment delivery if deemed appropriate in the best interest of the patient. Such interruption will be recorded in the eCRF. In case of machine breakdown or bank holidays, all measures will be taken to avoid prolonging the overall treatment time.

### Study procedures and participant timeline

The overview of study assessments and procedures is presented in Table 1.

**Table 1:** Assessment schedule

TIMEPOINT	Screening/ baseline	Treatment period		Follow-up period									
Window	W-4 à W0 Prior SBRT	W2		W3	1 month	3 months	6 months	9 months	12 months	15 months	18 months	21 months	24 months
Day	−28 to −1	8	12 ± 1	14 ± 3	30 ± 5	±10	± 10	±10	±10	±10	±10	±10	±10
Informed consent	X												
Medical history and demographic	X												
Prior / concomitant medication	X	X	X	X	X								
Tumor tissu and HPV Status	X												
Pregnancy test^a^	X												
Physical exam, weight	X	X	X	X	X	X	X	X	X	X	X	X	X
Performance status OMS	X	X	X	X	X	X	X	X	X	X	X	X	X
Dental examination and adapted care	X												
ORL exam *(mouth, throat and neck)*	X				X	X	X	X	X	X	X	X	X
Tumor bed evaluation *(clinical +/− nasofibroscopy)*	X				X	X	X	X	X	X	X	X	X
Adverse events													
*Acute toxicity*		X	X	X	X	X							
*Late toxicity*							X	X	X	X	X	X	X
Cervico-facial CT or MRI						X			X				X
thoracic CT^b^	(X)					X			X				X
PET-CT^c^						(X)			(X)				(X)
Quality of Life EORTC QLQ-C30 + HN35	X				X				X				X
SBRT		6*6Gy, 3 days/week in 11 to 13 days											

^a^ within 14 days before the start of stereotaxic radiotherapy

^b^To be repeated at inclusion only if the time between the preoperative thoracic scanner and the start date of radiotherapy is more than 3 months

^c^In case of local, regional, locoregional and / or distant recurrence, patients will be followed only for vital status

Patients are registered before SBRT is started.

#### Treatment (SBRT) period

Three visits with a physical evaluation will take place during radiotherapy: at the first fraction (D1), the fourth (expected date: D8) and the last fraction (D11 to D13). A visit at the end of treatment will be planned 7 days after the last fraction.

#### Post treatment period – follow up: 24 months after SBRT

After SBRT treatment, a visit will be planned at 1 week after the last fraction, at 1 month, at 3 months and then every 3 months (6 months, 9 months, etc., after SBRT) during the 2 years following SBRT. A head and neck and chest CT-scan will be performed at 3 months and at 1 and 2 years. MRI and/or FDG-TEP imagining assessments are left to the discretion of the investigators at each center.

### Statistical analysis

#### Sample size

For the determination of sample size, two parameters have been taken into account: 2-year severe late toxicity (grade ≥ 3) (primary end point) and two-year local control rate (secondary endpoint).

Postoperative SBRT efficacy and toxicity profiles are expected at least equivalent to usual treatments (brachytherapy and fractionated external beam radiotherapy; cf. Introduction for details).

For the Fleming’s single-stage model, we decided to accept a rate of severe late toxicity of less than 5% and to reject a rate greater than 15%. With a one-sided significance level test (α = 0.05) with 90% power (β = 0.10), the minimum of patients to accrue is 67. Postoperative SBRT will be considered unacceptable if 6/67 (9%) or more patients present 2-year grade ≥ 3 toxicity. Concerning the 2-year local control rate (secondary endpoint) we decided to reject a rate of less than 80% and to accept a rate higher than 90% (cf Rationale 2 for details). With a one-sided significance level test (α = 0.05; β = 0.20), the minimum of patients to accrue is 83. A less conservative assumption has been considered for statistical power for the 2-year local control rate which is a secondary endpoint. Postoperative SBRT will be considered as acceptable if 72/83 (87%) or more patients exhibit local control at 2 years. With these results and to compensate for eventual premature withdrawal, 90 patients will be included.

Because the main objective of this trial is evaluated at 2 years, once 30 patients (33% of accrual) are recruited, a review of all grade 3 and 4 toxicities will be communicated to the Independent Data Monitoring Committee (IDMC). The rate of grade 4 toxicity is expected to be inferior to 10%. Accrual will not be stopped during the evaluation of this parameter.

#### Data analysis

All analyses will be performed according to the International Conference on Harmonisation’s Good Clinical Practice guidelines. Categorical data will be described using counts per category and proportions with their 95%-CI. Quantitative data will be described using their mean, standard deviation and 95%-CI, and if their distribution is not Gaussian, by their median, quartiles and range, according to statistical distribution. The assumption of normality will be accessed using a Shapiro-Wilk test. The primary analysis will be performed in intention-to-treat. Objectives concerning survival extension (local control, locoregional control, DFS and OS) will be evaluated using Kaplan-Meier’s method. The confidence interval of survival rates will be calculated using the Rothman method.

An analysis of predictive factors will be conducted in order to identify clinical and/or dosimetric factors associated with differences in 2-year severe toxicity. In a multivariate context, predictive factors will be determined by a backward and forward stepwise approach applied to a logistic regression model. The covariates will be retained according to univariate results and clinical relevance.

Health-related QoL is assessed by two validated questionnaires (EORTC QLQ-C30 and HN35) at several time-points. The longitudinal analysis of QoL variations will be tested by random-effects models, useful to take into account between and within patient variability.

Because this trial is exploratory, no type-I error correction will be applied for multiple comparisons. Tests will be two-sided and *p*-values < 0.05 will be considered significant. A sensitivity analysis will be performed to measure the possible impact of missing data and to propose the most appropriate imputation approach.

### Data management and monitoring

An eCRF based on the Web-Based Data Capture (WBDC) system “Cleanweb” will be used for data collection, data management and monitoring. Health-related personal data captured during this study are strictly confidential and accessible only by investigators and authorized personnel. The investigator ensures the accuracy, completeness, and timeliness of the data recorded (pseudonymized patient data) and of the provision of answers to data queries.

The respect of the study protocol and procedures therein, and the quality of the collected data (accuracy, missing data, consistency with the source documents) will be regularly reviewed by site monitoring and central data monitoring. Monitoring reports will ensure traceability.

### Independent data monitoring committee (IDMC)

IDMC will review all safety problems or other issues identified during the medical review and seek advice as needed. Experts on the IDMC performing this review will be selected to have the relevant clinical trials/medical expertise. The committee will include 2 radiation oncologists and a statistician. Once 30 patients (33% of accrual) are recruited in the trial, the IDMC will be charged with reviewing all grade 3 and 4 toxicities.

## Discussion

This study is the first worldwide study to prospectively evaluate head and neck cancer postoperative SBRT for early-stage oropharyngeal or oral cavity carcinomas with high risk margins. Head and neck SBRT has been mainly studied in case of reirradiation [23–25, 28–30]. Lartigau et al. [28] reported the outcomes of a phase II trial including 56 patients treated with a dose of 36 Gy in 6 fractions over 2 weeks with Cyberknife™. The 3-month tumor response rate was 58% and the 1-year overall survival was 48%. Concerning acute toxicities, 4/56 patients had grade ≥ 3 mucositis, and 3/56 patients had grade ≥ 3 dysphagia. SBRT in head and neck cancers has also been evaluated as a boost after initial IMRT to the primary tumor and neck [26, 27]. Al-Mamgani et al. [26] studied SBRT as a treatment option for the boost of oropharyngeal cancers not suitable for brachytherapy. Fifty-one patients received boosts of 3 times 5.5 Gy after 46 Gy of IMRT to the primary tumor and neck. The treatment was well tolerated, as there were no treatment breaks and no grade 4 or 5 toxicity reported, either acute or chronic. The overall 2-year cumulative incidence of grade ≥ 2 late toxicity was 28%. Of the patients with 2 years with no evidence of disease (*n* = 20), only 1 patient was still feeding-tube dependent and 2 patients had grade 3 xerostomia. Concerning hypofractionated postoperative radiotherapy in head and neck cancers (reirradiation excepted), its use has already been reported in mucosal melanoma [34–36]. Wu et al. [36] reported in 2010 the outcomes of 27 patients (10 of them treated with 5 × 6 Gy over 2 weeks). 3/27 patients had grade 3 acute toxicities (2 epidermitis and 1 mucositis). With a 4-year median follow-up, no patients had grade ≥ 3 late toxicity.

One of the limitations of this study is that it is not a randomized trial. The justification not to run a randomized trial are as follows. First, there is no standardized control group radiotherapy treatment, as both brachytherapy and external beam radiotherapy can be applied. Choosing either brachytherapy or external beam radiotherapy as a control group would limit the number of recruiting centers, which might impair the feasibility of the project in a reasonable time period. Also, this is a relatively rare situation with relatively low recruitment capacities in a reasonable time period.

In this study, we hypothesize that the safety and efficacy profiles of postoperative SBRT for early-stage oropharyngeal or oral cavity carcinomas with high risk margins will be similar to other radiotherapy techniques (brachytherapy or fractionated IMRT) [12–21]. Thus, this technique could become a third alternative option along with fractionated external beam IMRT and brachytherapy. SBRT is an innovative radiotherapy technique that will likely be increasingly used in the future, as will other extracranial stereotactic techniques. This technique is now possible due to the rapid spread of dedicated stereotactic radiotherapy accelerators but needs to be properly regulated.

## Data Availability

The datasets generated during the current study are not publicly available since they will contain patient data and the informed consent agreement does not include sharing data publicly. The results of the trial will be published in peer-reviewed journals or disseminated through national and international conferences.

## Abbreviations

BED: Biologically effective doses
CTCAE: Common Terminology Criteria for Adverse Events
CTV: Clinical Target Volume
DFS: Disease-free survival
ECOG: Eastern Cooperative Oncology Group
GORTEC: Groupe d’Oncologie Radiothérapie Tête Et Cou
HPV: Human papillomavirus
IDMC: Independent Data Monitoring Committee
IMRT: Intensity-modulated radiation therapy
LDR: Low dose rate
OAR: Organs at risk
OS: Overall survival
PDR: Pulsed dose rate
PRV: Planning organ at risk volume
PTV: Planning target volume
QoL: Quality of life of patients
SBRT: Stereotactic radiotherapy
VMAT: Volumetric modulated arc therapy
WBDC: Web-Based Data Capture
